# Sol-Gel Material-Enabled Electro-Optic Polymer Modulators

**DOI:** 10.3390/s150818239

**Published:** 2015-07-27

**Authors:** Roland Himmelhuber, Robert A. Norwood, Yasufumi Enami, Nasser Peyghambarian

**Affiliations:** 1 College of Optical Sciences, The University of Arizona, 1630 E. University Blvd., Tucson, AZ 85721, USA; E-Mails: rnorwood@optics.arizona.edu (R.A.N); nasser@optics.arizona.edu (N.P); 2 School of System Engineering, Kochi University of Technology, 185 Miyanoguchi Tosayamada, Kochi 782-8502, Japan; E-Mail: enami.yasufumi@kochi-tech.ac.jp

**Keywords:** sol-gel, EO polymers, modulators

## Abstract

Sol-gels are an important material class, as they provide easy modification of material properties, good processability and are easy to synthesize. In general, an electro-optic (EO) modulator transforms an electrical signal into an optical signal. The incoming electrical signal is most commonly information encoded in a voltage change. This voltage change is then transformed into either a phase change or an intensity change in the light signal. The less voltage needed to drive the modulator and the lower the optical loss, the higher the link gain and, therefore, the better the performance of the modulator. In this review, we will show how sol-gels can be used to enhance the performance of electro-optic modulators by allowing for designs with low optical loss, increased poling efficiency and manipulation of the electric field used for driving the modulator. The optical loss is influenced by the propagation loss in the device, as well as the losses occurring during fiber coupling in and out of the device. In both cases, the use of sol-gel materials can be beneficial due to the wide range of available refractive indices and low optical attenuation. The influence of material properties and synthesis conditions on the device performance will be discussed.

## 1. Introduction

Sol-gel materials are most commonly discussed as either sols, gels, resins or films, which are prepared by the hydrolyzation-condensation reaction of metal alkoxides or halides. A sol is a subclass of colloid, which is defined as a suspension in which the suspended particles are so small, that gravity has little influence on the position or movement when compared to Brownian motion [1]. Common colloids are fog, where water droplets are suspended in air, and milk, where fat and protein droplets are suspended in water. If a solid is suspended in a liquid, the suspension is called a sol. If the sol is dried and the condensation reaction continues until the formed molecules grow larger and larger, combining into virtually a single molecule with the macroscopic dimensions of the reaction vessel, the material is called a gel. If, during solvent removal, the sol particles do not react with each other and stay separated, the resulting liquid is often called a resin. It is, however, not necessary to remove the solvent for gelation to occur. A solvent-free resin can then be either thermally- or UV-cured if the appropriate moieties (like an organic epoxy or methacrylate group) are present. This can be done in the bulk or after film formation by spin or dip coating. It is also possible to spin film directly from a sol; the formed film is technically also a gel, but often more dense.

The sol-gel materials discussed in this article are mostly also inorganic organic hybrid materials [2]. This means that organic and inorganic parts of the material are mixed on a molecular level. Organic molecules are commonly described as consisting mainly of carbon and hydrogen. C-C bonds form the backbone of molecules and polymers, while hydrogen populates the bonds not occupied by carbon. Sulfur, oxygen and nitrogen can be present, among others, as well. Inorganic compounds can be classified in many different ways; classes include oxides, metals, minerals and ceramics. Inorganic-organic hybrids are materials in which the organic and inorganic parts are intertwined.

**Figure 1:**
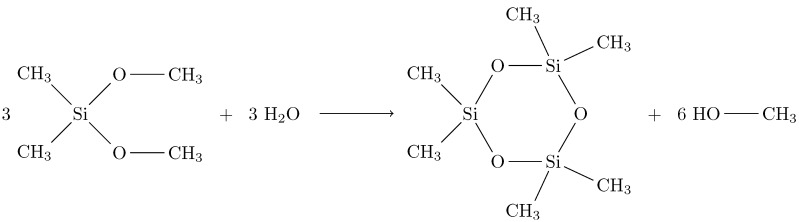
Hydrolyzation condensation reaction of dimethyl dimethoxy silane.

A very simple case of this is shown in Figure 1. Here, dimethyl dimethoxy silane reacts with water to form a ring of silicon and oxygen. The ring itself can be considered inorganic, because it only contains silicon (a half metal) and oxygen. The methyl groups attached to the silicon, on the other hand, are purely organic.

A benefit of using inorganic-organic hybrid materials is that the material properties can be altered easily by choosing precursor molecules with different organic end groups, such that the resulting material can be designed to meet the needs of the specific application. The synthesis of these sol-gel materials is typically a one-pot single-step process, which can be done in a simple scintillation vial without the need for chemical glassware and numerous reaction setups.

**Figure 2:**
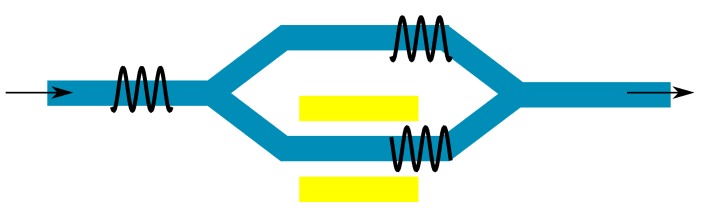
Schematic of a Mach–Zehnder modulator. The phase of the modulated arm is changed by *π*.

In general, an electro-optic (EO) modulator transforms an electrical signal into an optical signal. The incoming electrical signal is most commonly information encoded in a voltage change. This voltage change is then transformed into either a phase change or an intensity change in the light signal. In principal, light has two more degrees of freedom, namely wavelength and polarization, which are used for multiplexing, but not as commonly used in modulation schemes. As EO materials change their refractive index if a voltage is applied across them, intensity modulation is also based on phase modulation, via constructive or destructive interference. The most common device design for this is a Mach–Zehnder modulator (MZM); a schematic is shown in Figure 2. When voltage is applied to one arm of the MZM, the refractive index of the material changes. If the phase of the light passing through this arm is either enhanced or retarded by *π*, the modulated light and the non-modulated light interfere destructively. The voltage at which this happens is called $V_{\pi }$.

The most commonly-used material for EO modulators is lithium niobate (LiNbO${}_{4}$). Lithium niobate is a non-centrosymmetric crystal and, therefore, has a non-zero $\chi^{\left(2\right)}$ tensor. The corresponding $r_{33}$ coefficient is around 32 pm·V${}^{-1}$ [3] at 1550 nm, where the ordinary refractive index is 2.13 and the extraordinary refractive index is 2.21 [4]. Waveguide fabrication is commonly done by titanium in-diffusion processes [3] or proton exchange [5].

Silicon-based modulators based on plasma dispersion [6] have been demonstrated some time ago, and this is now, together with carrier depletion, the most widely-used approach. The $V_{\pi }$ L for a Mach–Zehnder configuration is around 1.4 Vcm up to 12 GHz [7]; for a micro-ring geometry, switching voltages are 1 V or lower [8]. If photonic crystal structures are incorporated to make use of the slow light effect, $V_{\pi }$ L values as low as 0.05 Vmm with 20 dB total insertion loss have been shown operating up to 2 GHz [9]. Liquid crystals can also be used for electro-optic modulators [10,11]. A more comprehensive review of EO modulators was published recently [12].

EO guest-host polymers consist of an amorphous polymer matrix and a dye with a permanent dipole moment. These mixtures are most often prepared in solution and then spun cast into films; after spin casting, the dye molecules are oriented randomly. During the poling process, a high electric field is applied, and the film is heated up close to the glass transition temperature of the polymer. The polar dye molecules align along the electric field lines, creating a non-centrosymmetric order, which is frozen in as the material is returned to room temperature with the field still applied. As such, the now poled polymer has a non-zero $\chi^{\left(2\right)}$ tensor and exhibits the electro-optic effect, usually quantified in terms of the Pockels coefficient. A second order nonlinear optical material can also be used to create second harmonic light, and the intensity of the generated second harmonic depends quadratically on the *d* coefficients, which can be directly related to the Pockels coefficient [13].

Poled polymer films, which exhibit second order nonlinear optical properties, have been investigated for over 20 years [14]. Optical polymers are, aside from technically impractical single-crystal examples, amorphous materials. To show second-order nonlinearities, some sort of order has to be introduced into the system, such as via the poling process described above [15].

EO polymers have been successfully used in EO modulators showing sub-volt $V_{\pi }$ [16] (1.8 Vcm $V_{\pi }L$), low optical loss [17] and bandwidths above 100 GHz [18]. To date, the best available crosslinkable EO polymers exhibit a combination of both large $r_{33}$ values of 100 pm·V${}^{-1}$ and excellent thermal stability up to 230 ${}^{\circ }C$, a very important advance that can enable high-performance organic EO materials to fulfill the CMOS compatibility requirement [19].

There are also numerous approaches to integrating EO polymers on the silicon platform, such as silicon slot waveguides [20,21,22] and photonic crystal waveguides [22]. Slotted waveguide modulators have $V_{\pi }$ L below 1 Vcm, but optical insertion losses above 35 dB. $V_{\pi }$ L below 0.5 Vcm with losses around 22 dB [23] has been achieved with photonic crystal structures.

**Figure 3:**
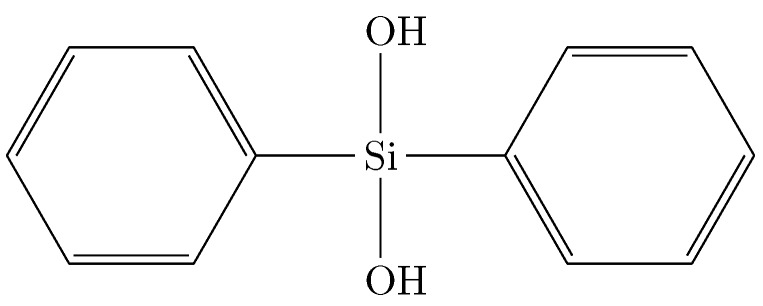
Diphenylsilanediol.

Sol-gel materials have been used in the development of electro-optic (EO) polymer-based modulators [24,25], because of their variety of available refractive indices. They can be used to form passive waveguide transitions to minimize coupling loss or as cladding layers for the EO polymer [17]. The demands for these two applications are different. For waveguide transitions, the most important factor is the optical loss and the accessibility of a large refractive index range. If the sol-gel material is used as a cladding for the EO material, the electrical conductivity at poling conditions, as well as the dielectric constant are important factors for efficient poling and driving the modulator. Polymer-based EO modulators have been of great interest for a number of years, mainly because of their high speed capabilities. Much effort has been put into the development of polymeric materials with very high $r_{33}$ coefficients, as well as in the overall device design [26]. Photopatternable sol-gel materials with low (<0.8 dB·cm${}^{-1}$) losses at 1.55 m have been known for some time. They were first reported via a water-free synthesis [27]. This method uses a prehydrolyzed precursor, diphenylsilanediol (see Figure 3), which reacts under base catalysis with a crosslinkable alkoxysilane. Because no additional water is introduced into the synthesis, the percentage of uncondensed Si-OH groups is negligible. This eliminates one major source of absorption losses common in sol-gel materials, *i.e*., large absorption by the OH functionality.

The use of sol-gel materials for increased poling efficiency has been demonstrated, but the reason for it is much less well understood. We will therefore not attempt to give a completely mechanistic view of the reported results, but rather give examples that support certain ideas that can be used to improve poling efficiency.

## 2. Material Loss in Sol-Gel Materials

Absorption losses in the telecom O and C band (1300–1600 nm) in organic materials is caused by molecular vibrations. Specifically, the overtone vibrations of C-H, OH and N-H are of concern [28,29]. A table with the absorption peaks of different chemical species can be found in Table 1 [30].

**Table 1:** Absorption bands in the near-infrared.

Wavelength (nm)	Assignment
1150	CH[CH${}_{3}$] third overtone
1200	CH[CH${}_{2}$] third overtone
1380	CH[CH${}_{3}$]+*δ* SiOSi second overtone
1420	CH[CH${}_{2}$]+*δ* SiOSi second overtone
1300–1600	Si-OH and Zr-OH
1630	CH[CH=CH] second overtone

A water-free synthesis is extremely useful for eliminating loss caused by OH groups, but it restricts the number of available precursors, because diphenylsilanediol is the only commercially available silanediol. It is however possible to reduce the optical losses through aqueous routes [31,32]. This places no restrictions on the precursors, but creates the need for a very well-controlled hydrolysis-condensation reaction to ensure a minimum of remaining OH. Specifically, the amount of water used in the reaction plays a major role [32]. If the amount of water is too large, not all hydrolyzable groups will be condensed, and left over OH will be present. If the amount of water is too small, insufficient formation of the inorganic network will occur, which can lead to poor mechanical properties. The large number of left over alkoxy groups also increases the loss because of the CH${}_{2}$ and CH${}_{3}$ groups in the alkoxy functionality, which result in absorption in the NIR because of the CH stretch vibration. Most water-based sol-gel materials for waveguide applications also contain zirconium alkoxides to increase the adhesion of the material to the substrate. However, the presence of zirconium leads unfortunately to the absorption of water in the cured film [33], as well as to a reduction in shelf life. It can however also lead to lower losses, because the very reactive zirconium species will immediately react with any available OH groups [32]. The wide availability of silane precursors makes it easy to tailor the properties of sol-gel materials to fit the needs of the application. The ability to cure a material by UV or to photo pattern it, respectively, is a very important feature of a material, as it makes the fabrication of waveguide devices much easier. A common way of achieving this with sol-gel materials is to use a precursor with an organic polymerizable group. The most common molecule for this is methacryloxypropyltrimethoxysilane (MAPTMS). The chemical structure of MAPTMS, as well as the structures of other silanes used can be found in Table 2 [31]. The optical loss at telecom wavelengths for some of the precursors have been measured to be below 0.5 dB·cm${}^{-1}$ [34]. MAPTMS is used as in polymers to increase adhesion [35], as well as a surface modifier to increase adhesion [36]. Because MAPTMS has a large proportion of aliphatic bonds, the amount of MAPTMS should be reduced if low propagation loss is required (see Table 1). diphenyldimethoxysilane (DPDMS) has a higher base refractive index than MAPTMS, and therefore, adding it to a MAPTMS-containing sol-gel material will increase the refractive index. After full condensation, DPDMS does not contribute any aliphatic bonds, so optical loss can be reduced, as well. For the same reasons, phenyltrimethoxysilane (PTMS) can be used [37]. To reduce the refractive index, a precursor with a lower refractive index than MAPTMS should be used. Fluorinated organic molecules show a lower refractive index than their non-fluorinated counterparts [38]. Perfluorinated siloxane precursors have been used to reduce the optical loss of sol-gel materials dramatically [39]. The photo-sensitivity of fluorinated materials is reduced, however, due to the high permeability of fluorocarbons to oxygen [40] and, therefore, higher oxygen inhibition of the radical polymerization reaction [41]. Zirconium(IV) propoxide (ZPO) is widely used [42] to change the refractive index of sol-gel materials for waveguide fabrication [43], to reduce the optical loss [32,33] and to improve mechanical stability after UV curing [44,45]. As a Q-system with four hydrolyzable groups, ZPO can, however, in large quantities lead to gelation prior to spin coating.

**Table 2:** Silane precursors used to tune the refractive index and to reduce the optical loss. Zirconium(IV) propoxide (ZPO) is only stable as a solution in propanol, so no refractive index is given.

Chemical Name	Chemical Structure	$n_{D}^{20}$ [46]	Benefits	Drawbacks
Methacryl- oxypropyl- trimethoxysilane (MAPTMS)	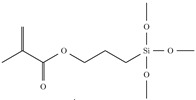	1.4277	Better adhesion, photo-patternable	increases loss
Diphenyl- dimethoxysilane (DPDMS)	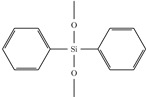	1.5447	increases index, decreases loss	
Phenyl- trimethoxysilane (PTMS)	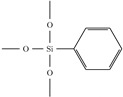	1.4734	increases index, decreases loss	
Bis-pentafluoro- diphenyl- dimethoxysilane (BPFDPDMS)	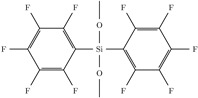	1.4181	lowers index, decreases loss	reduces photo-sensitivity
			
Zirconium(IV) propoxide (ZPO)	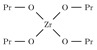	n/a	increases index	increases viscosity, can cause gelation

**Figure 4:**
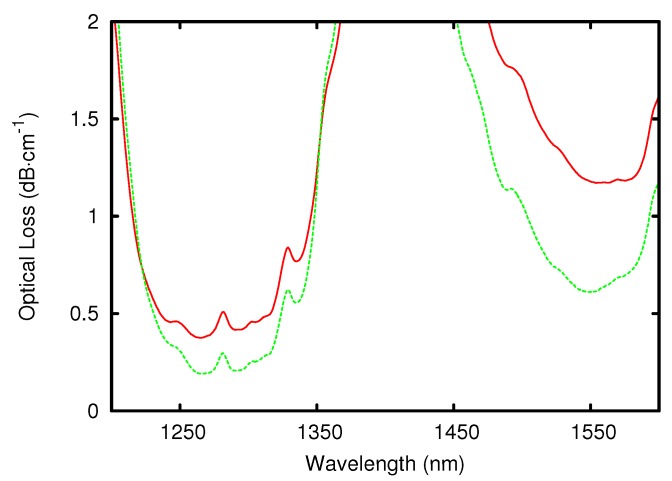
Absorption spectra of two example systems with hydrolyzation ratios of 0.45 (solid red) and 0.36 (dashed green), respectively, are shown. The large absorption peak between 1350 and 1450 nm is due to bond vibrations between carbon-carbon and silicon-oxygen (see Table 1).

**Figure 5:**
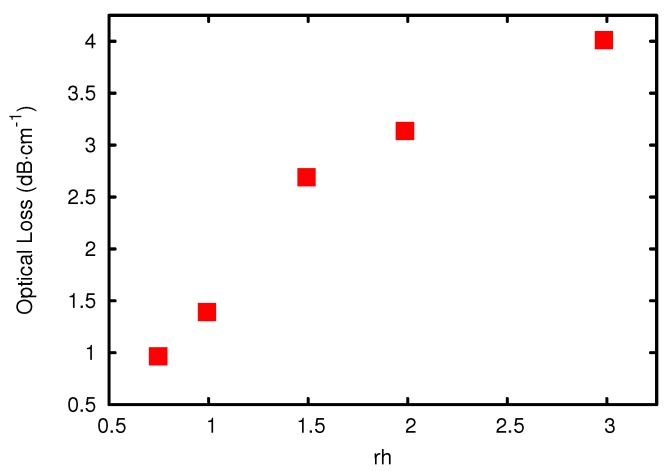
Optical loss at 1550 nm *vs.* the amount of water used for hydrolysis.

One factor influencing the optical loss of a system in the NIR is the hydrolyzation ratio (rh), which is the ratio of added water to the number of hydrolyzable alkoxy groups. For every hydrolyzed alkoxy group, two alcohols are produced, one in hydrolysis and one in condensation, during which the Si-O-Si bond is formed. If the condensation is carried out between a hydrolyzed and non-hydrolyzed species, the alcohol is separated directly. If the condensation is carried out between two hydrolyzed species, water instead of alcohol is formed, which can afterwards again hydrolyze an alkoxy group. The amount of water added to the synthesis determines the amount of hydrolyzation and condensation and, therefore, the average size of the resulting oligomers. The larger these oligomers are, the higher the viscosity of the resulting material will be. Because the higher the condensation ratio the denser the resulting network, the amount of water also influences the refractive index of the material slightly. The influence of the hydrolyzation ratio on the optical loss of the resulting materials in the telecom regime at 1550 nm is of great interest. It was found that by decreasing the hydrolyzation ratio from 0.45 down to 0.36, the optical loss can be drastically decreased from 1.2 dB·cm${}^{-1}$ to 0.6 dB·cm${}^{-1}$ [31]. The measured spectra can be seen in Figure 4. Both systems had a molar ratio of 1:1 MAPTMS:DPDMS and used 0.1 molar hydrochloric acid (HCl) as a catalyst. The reason for the strong influence of the hydrolyzation ratio on the optical loss is that some hydrolyzed groups are not accessible for the condensation reaction as the oligomers grow around them and, therefore, form a protecting shell. The hydrolyzation ratio must therefore be chosen to minimize the amount of these inaccessible SiOH groups, but still large enough to result in complete hydrolysis. The influence of the hydrolyzation ratio on the loss of a ZPO containing system can be seen in Figure 5. It was also observed that adding less water creates a sol that is less viscous and therefore produces thinner films and that films made from sol-gel with an rh of less than 0.9 were too soft for use as a cladding for EO polymers [47].

**Figure 6:**
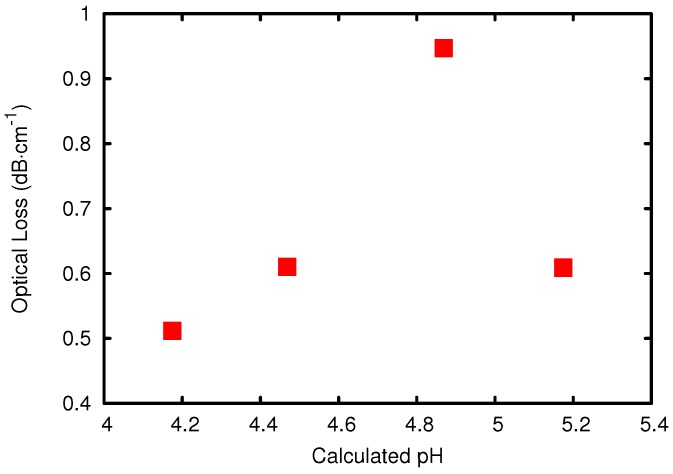
Optical loss of the resulting material over the initial pH of the reaction mixture.

The influence of the amount of catalyst on the optical loss was also examined [31]. For this synthesis, the same system as described above (1:1 MAPTMS:DPDMS) was used with a hydrolyzation ratio of 0.36. The pH was varied by using different solutions of hydrochloric acid. The used hydrochloric acid solutions were 0.1 M, 0.2 M, 0.5 M and 1 M, respectively. The optical loss plotted *vs*. the pH of the reaction mixture can be seen in Figure 6. The pH is technically not defined in non-aqueous solutions, so the pH values given relates to the calculated H${}_{3}$O${}^{+}$ concentration in the reaction mixture.

## 3. Adjusting the Refractive Index in Sol-Gel Materials

**Figure 7:**
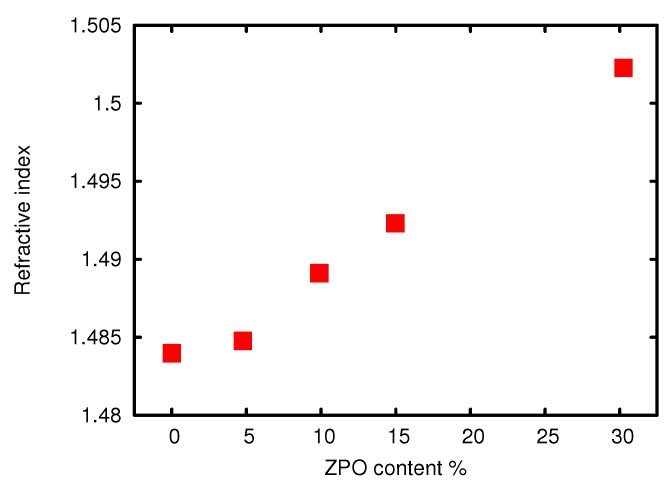
Refractive index at 1550 nm *vs*. mol % of ZPO added to MAPTMS.

**Figure 8:**
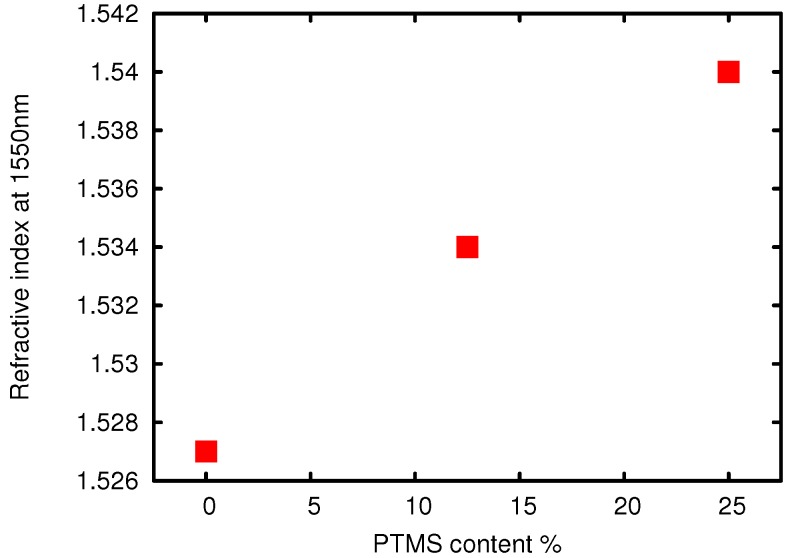
Refractive index at 1550 *vs*. mol% of PTMS, measured in cured films.

Since the sol-gel materials are to be used in planar optical waveguides, it is quite useful to be able to change the refractive index of the material easily, without changing the other properties drastically. The most common way to tune the index of sol-gel materials is to vary the amount of zirconium (IV)-propoxide added during the synthesis. The resultant change in the refractive index can be seen in Figure 7. The linear variation of the refractive index with the addition of ZPO is suggestive of the fact that the Zr is incorporated directly into the silicon network and does not aggregate. An alternative method for changing the refractive index, chosen here, is to introduce silanes as index changing agents. If a material with a molar ratio of 1:1 MAPTMS:DPDMS is chosen as the cladding material for optical waveguiding, the material used for the core must have a higher index, and this was accomplished by replacing a certain fraction of MAPTMS with phenyltrimethoxysilane (PTMS). The influence of the PTMS content on the refractive index is, as expected at these low molar percentages, very linear and can be seen in Figure 8. It is even possible to increase the refractive index up to 1.562 if naphthalene trimethoxysilane (NaphTMS) is used instead of PTMS. Another way of achieving this high index is by substituting MAPTMS with styryltrimiethoxysilane(SETMS). Lowering the refractive index of a given material can be achieved by substituting aromatic compounds (see Table 2) with perfluorinated ones, like bis-pentafluorodiphenyldimethoxysilane(BPFDPDMS).

## 4. Sol-Gel Materials for Improved Poling

In a waveguide modulator, the EO polymer is the core layer and is surrounded by an upper and lower cladding. Therefore, the modulator must be poled with at least the lower cladding present.

An ideal cladding material would have several important properties. Its conductivity should be higher than the EO polymer, so that there is little voltage drop over the cladding region. The dielectric constant should be higher than that of the EO polymer, causing the *E*-field to be enhanced in the EO region, and finally, it should suppress Fowler–Nordheim tunneling [48] to prevent fatal brake down.

Sol-gel materials containing MAPTMS and ZPO have been shown to increase the poling efficiency of EO polymers [49]. DeRose *et al.* found that the measured current density as a function of the applied electric field for the sol-gel cladding layer alone was observed to be between one to two orders of magnitude greater than that of the EO polymers for applied fields between 40 and 120 Vm ${}^{-1}$, and poling efficiency was improved by 20%.

They concluded that the sol-gel cladding has a much larger conductivity than the EO polymer layers at the poling temperature, which should be ideal for efficient multilayer poling. Conducting polymers (PDOT/PMMA) have also been used to improve poling efficiency [50]; other groups have used Baytron P [50,51]. The improvement in poling efficiency was between 20 and 30%. A recent paper linked increased poling efficiency to increased conductivity of the MAPTMS/ZPO sol-gel bottom cladding layer [53]. Here, a one order of magnitude increase in conductivity of the sol-gel layer increased the poling efficiency by more than 100%. One would therefore conclude that the higher conductivity of the cladding layer should lead to higher poling efficiency.

**Figure 9:**
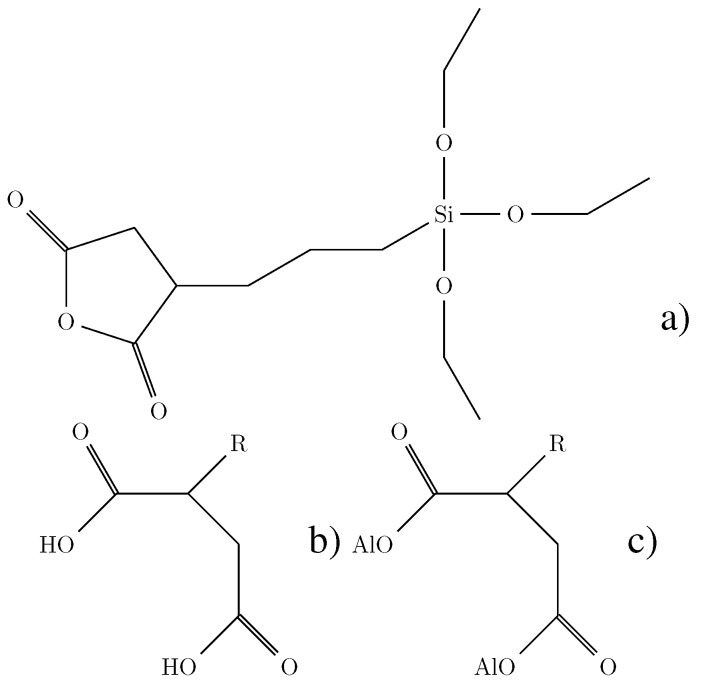
Structure of 3-(triethoxysilyl)-propyl succinic acid anhydride (**a**), the di-acid (**b**) and a di-ester (**c**). Al stands for an alkyl group.

The conductivity of sol-gel materials without any zirconium can be increased by the addition of methacrylic acid. The amount of methacrylic acid is basically limited by the reduction of film quality and adhesion that results with its addition. The maximum amount of methacrylic acid that produced a useful material was about 3.5%. Another approach to introducing acid functionalities is to form them *in situ* during the sol-gel reaction. As a source for carboxylic acid functionality, 3-(triethoxysilyl)-propyl-succinic-acid-anhydride (SucAHTES) can be used [54]. The structure can be seen in Figure 9.

The anhydride ring can open up under acidic or basic condition to form the di-acid. Another product is the ester, which can be formed from the acid stage or the anhydride stage. To minimize the formation of the ester, the hydrolyzation of the anhydride and the formation of the inorganic-organic network were carried out in two stages. To reduce the concentration of methanol and ethanol from the sol-gel reaction, iso-propanol was chosen as a solvent. Iso-propanol is sterically hindered and will not form esters easily. The ester does not contain any easily-detachable protons and, therefore, does not contribute to the conductivity. The structures of the di-acid and the di-ester are also shown in Figure 9. To measure the conductivity of the material, samples with indium tin oxide (ITO) as a bottom electrode and gold as a top electrode were prepared. The materials were spun coated and cured by UV exposure. At an applied field of 50 Vcm${}^{-1}$, the samples where heated in a inert gas atmosphere at approximately 13 ${}^{\circ }C$ min${}^{-1}$–150 ${}^{\circ }C$ . The current was recorded by using a picoammeter in series with the sample. The general behavior of all samples was a steady increase in current with a slight plateau around 110 ${}^{\circ }C$ . The highest current was observed at 150 ${}^{\circ }C$ . The observed current densities were almost 10-times higher than reported by DeRose *et al*. [54]. However, no increase in poling efficiency with the highly conducting materials was observed with several different EO polymers, leading to the conclusion that conductivity is not the only parameter that needs to be considered here.

It seems that also the kind of conduction mechanism plays a role in poling. In the case of ZPO-containing sol-gel materials, conductivity is probably due to water absorption [33]. The absorbed water can act as a matrix to allow positive ions to flow in both directions. In the case of conducting polymers, like PDOT, the conductivity is electronic and caused by long chain conjugation [55]. In the SucAHTES-based materials, the conductivity is most likely only hole or proton-based, similar, but much weaker than in sulfonyl group-containing materials [56,57].

**Figure 10:**
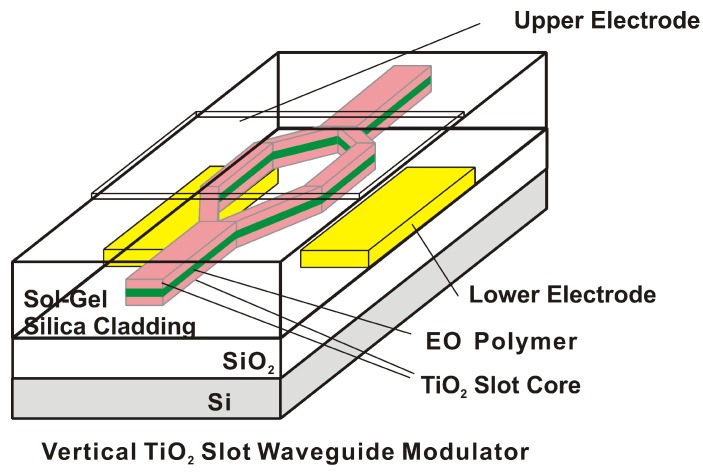
EO polymerTiO${}_{2}$ vertical slot waveguide modulator [59].

Sol-gel-derived titanium dioxide has been shown to improve the poling efficiency by 30–40% [58], and these studies identified field distribution flattening effects as the major factor in enhancing the poling efficiency of EO polymers. Due to its low lying valence band, the TiO${}_{2}$ layer can effectively block hole injection from the anode and prevent dielectric breakdown due to Fowler–Nordheim tunneling [58]. Titanium dioxide can be incorporated into devices in multiple ways; an example is an EO polymer/TiO${}_{2}$ multilayer slot waveguide modulator. This device was fabricated using double TiO${}_{2}$ thin-film layers on the sol-gel silica cladding, MAPTMS, as shown in Figure 10 [59]. The EO polymer/single TiO${}_{2}$ waveguide modulator incorporated a low-index mesoporous sol-gel silica cladding [60] to increase mode confinement in the EO polymer. The mesoporous sol-gel silica cladding was fabricated as a template method with a surfactant, in which tetraethyl orthosilicate (TEOS) was calcined at 400 ${}^{\circ }C$ for three hours to remove the surfactant and to construct the mesoporous structure in the sol-gel silica cladding. The refractive index of the mesoporous sol-gel silica was 1.21–1.26 at a wavelength of 800 nm, as determined using ellipsometry. In the integrated optical modulators, we used a low-index EO polymer SEO125 (35 wt% chromophore doped in amorphous polycarbonate, index of 1.621 at 1550 nm) to obtain higher mode confinement in the EO polymer between two TiO${}_{2}$ slot layers. The waveguiding mode was calculated, using the three-dimensional finite differential time domain (3D FDTD) method. A mode confinement factor in the >400 nm-thick EO polymer was calculated to be 68 %, which was better than that in previous 300 nm-thick device [59].

The EO polymer contains chromophore has to be electrically poled at the glass transition temperature (e.g., 150 ${}^{\circ }$C) to have electro-optic (Pockels) effect in the modulator devices. Poling efficiency was improved when >400 nm-thick low-index EO polymer was poled with the sol-gel silica MAPTMS that was cured at 150 ${}^{\circ }$C for 0.75 hour. The poling conductivity for the multilayer (EO polymer/TiO${}_{2}$/sol-gel silica MAPTMS) was increased to $3.2\cdot 10^{-8}$ Sm${}^{-1}$, which was approximately 30-times that of the modulator having a 0.3 m-thick EO polymer poled with TiO${}_{2}$ and the sol-gel silica cladding calcined for two hours [53]. The in-device $r_{33}$ value was the highest for the low-index EO polymers SEO125 [61], because higher resistance of the EO polymer layer on the TiO${}_{2}$ layer enabled a higher poling field for the EO polymer. Y. Enami *et al.* successfully demonstrated lower $V_{\pi }$ for the EO polymer/TiO${}_{2}$ multilayer vertical slot waveguide modulator. From the transfer function between the voltage signal and the optical signal at 1 kHz, $V_{\pi }$ was measured to be 2.0 V for a 10 mm-long electrode ($V_{\pi }L_{e}$ = 2.0 Vcm, in-device $r_{33}$ = 78 pm/V) at a wavelength of 1550 nm [53]. Since low-index EO polymers, such as SEO125, usually have lower EO coefficients, Y. Enami *et al.* is now trying to employ high-index SEO100 [61] (refractive index = 1.705 at 1550 nm wavelength), which has in-device $r_{33}$ of 160 pmV-1 in their previous demonstration [62]. Y. Enami *et al.* investigated the electrical properties and optical quality of two layers, the TiO${}_{2}$ selective layer and the sol-gel silica cladding layer, for use as coating layers for EO polymers in EO polymer/TiO${}_{2}$ multilayer slot waveguide modulators. Y. Jouane *et al.* used a simple ellipsometric reflective technique developed by Teng and Man to measure the EO coefficients of poled thin films of the EO polymer SEO100 in an EO multilayer device.

It is difficult to pole 0.3–0.4 µm -thick SEO100 efficiently, because dielectric breakdown frequently happened at low poling voltages (e.g., 10 V) due to the non-uniform thickness of the EO polymer. As Jouane *et al*. mentioned [63], when 0.3–0.4 µm -thick SEO100 was poled with a 100 nm-thick TiO${}_{2}$ layer without sol-gel silica cladding, the applied electric field across the thin SEO100 was higher and exhibited electro trap-free conduction. The non-blocking electrical contacts increased the flow of charges, which enabled better distribution of the electric field across the SEO100. However, due to dielectric breakdown at low poling voltages, 0.3–0.4 µm -thick SEO100 could not be poled efficiently. Adding the sol-gel cladding into SEO100/TiO${}_{2}$ layers increased the breakdown voltage, because the tunneling current was significantly suppressed. Jouane *et al*. experimentally showed Schottky–Richardson thermionic emission and one order higher poling conductivity when the 0.3–0.4 µm -thick SEO100 was poled with TiO${}_{2}$ and sol-gel silica at 158 ${}^{\circ }C$ [63]. The poling field for SEO100 was estimated to be more than 400 V/µm , which resulted in the highest EO coefficient ever measured for this material (198 pm/V at 1550 nm). The EO coefficient was also measured to be 226pm/V using another laser at a 1.31-µm wavelength, which was explained well by the two-level model [64].

## 5. Conclusions

Sol-gel materials can help make better devices by reducing propagation and coupling loss. The material loss of sol-gel materials in the telecom region can easily be as low as 0.8 dB·cm${}^{-1}$, and the refractive index can be modified to allow for a wide range of designs. Sol-gel materials can improve the poling efficiency of electro-optic polymers due to field concentration in the polymer layer and reduced charge injection. The conductivity of the surrounding materials also plays a major role in the efficient poling of EO polymers, but some materials seem to be working much better than others. A possible explanation could lay in the conduction mechanism.
